# Knowledge mapping and global trends of drug hypersensitivity from 2013 to 2023: A bibliometric analysis

**DOI:** 10.1002/iid3.1245

**Published:** 2024-04-17

**Authors:** Li Luo, Niannian Chen, Zhanpeng Li, Chunmei Zhao, Yiming Dong, Likai Wang, Xiaoqian Li, Wenchao Zhou, Yingna Li, Cairong Gao, Xiangjie Guo

**Affiliations:** ^1^ Department of Pathology, School of Forensic Medicine Shanxi Medical University Taiyuan China; ^2^ School of Public Health, Academy of Medical Science Shanxi Medical University Taiyuan China; ^3^ First Clinical Medical College Shanxi Medical University Taiyuan China; ^4^ Translational Medicine Research Center Shanxi Medical University Taiyuan China

**Keywords:** bibliometric analysis, CiteSpace, drug hypersensitivity, knowledge domain visualization

## Abstract

**Background:**

Drug hypersensitivity is a major global public health issue with a significant increase in prevalence in populations. Here, we provide a deep insight into the frontier hotspot and future direction in the field of drug hypersensitivity.

**Methods:**

A knowledge map is portrayed based on publications related to drug hypersensitivity from Web of Science Core Collection using CiteSpace. Co‐occurrence relationships of countries, institutes, authors, journals, references, and keywords are constructed. According to the co‐occurrence relationships, hotspots and future trends are overviewed.

**Results:**

The United States ranked first in the world and China with the second highest publications was the only developing country. Torres, Mayorga, and Blanca were highly productive authors. Harvard University was the institution with the most research publications. Keywords co‐occurrence analysis suggested applications in emerging causes, potential mechanisms, and clinical diagnosis as the research hotspots and development frontiers.

**Conclusion:**

Research on drug hypersensitivity is in a rapid development stage and an emerging trend in reports of anaphylaxis to polyethylene glycols is identified. Developing algorithms for understanding the standardization process of culprit drugs, clinical manifestations, and diagnostic methods will be the focus of future direction. In addition, a better understanding of the mechanisms to culprit drugs with immunological precise phenotypic definitions and high‐throughput platforms is needed.

## 1 INTRODUCTION

Anaphylaxis is an acute systemic hypersensitivity reaction caused by an allergen that results in skin and mucous membrane damage, as well as respiratory and cardiovascular dysfunction.^1^ According to the World Allergy Organization's anaphylaxis guidance in 2020, a global incidence of anaphylaxis in humans is between 50 and 112 people per 100,000 people per year.^2^ What is more, a recent systematic review showed that the global incidence and prevalence of anaphylaxis in children ranged from 1 to 761 per 100,000 people per year.^3^

Food, insect venom, and drugs of anaphylaxis are the most common culprits worldwide, which are on the rise.^4^ When drug reactions resembling allergy occur, they are referred to as drug hypersensitivity until evidence of drug‐specific antibodies or T cells is shown.^5^ The prevalence of drug hypersensitivity is increasing, which is consistent with the general increase in the population's sensitivity to different allergens.^6^ Moreover, drug hypersensitivity is a major global public health problem on account of life‐threatening anaphylaxis and rare severe cutaneous reactions.^7^ Additionally, drug hypersensitivity is heterogeneous and their classification is challenging. Clinically, drug hypersensitivity can be classified as immediate drug hypersensitivity reactions (IDHR) and nonimmediate drug hypersensitivity reactions (NIDHR).^8^ IDHR (urticaria, angioedema, anaphylactic shock, and so on) occur within minutes or hours following drug exposure and are possibly induced by an immunoglobulin E (IgE)‐mediated mechanism. NIDHR (delayed urticaria, Stevens‐Johnson Syndrome, etc.) take several days or even weeks of initial drug administration and are mainly associated with a delayed T‐cell‐dependent type of allergic mechanism. Mechanistically, drug hypersensitivity can be divided into immune drug hypersensitivity mediated by IgE, IgG, and immune complex complement (referred to as allergic anaphylaxis), and nonimmune drug hypersensitivity mediated via vasoactive and other inflammatory factors released without the participation of a specific immune mediator (referred to as nonallergic anaphylaxis).^9^ Nowadays, drug hypersensitivity is becoming more complex with more drug classes commonly involved. Although many authors have published research findings on drug hypersensitivity, the management of drug hypersensitivity faces numerous challenges because of lacking standardized drug allergens and reliable diagnostic methods, as well as limited treatment manners. Hence, it is quite necessary to obtain data from drug hypersensitivity for assisting researchers in analyzing the evolution and emerging trends on the subject.

Bibliometric analysis refers to the quantitative and qualitative analysis of a comprehensive knowledge system in a specific field. It has been widely used to gain insight into the knowledge structure and global trends over time.^10^ Additionally, bibliometric analysis allows for identifying the collaborative relationships and academic contributions among authors, institutions, and countries. CiteSpace is a Java‐based software that has been commonly applied for bibliometrics analysis. Recently, more and more researchers have used CiteSpace to evaluate their respective research domains. On the knowledge mapping of drug hypersensitivity, however, no specific bibliometric analysis has been conducted to date.

## 2 METHODS

### 2.1 Data collection

The Web of Science Core Collection database (WoSCC) is the foremost research platform for hard science, social science, arts and humanities information, as well as an independent global citation database for the world's most trustworthy publisher. To improve data representation and accessibility, we searched articles in the Science Citation Index Expanded of WoSCC on July 1, 2023. The main search term was “Topic Search = drug AND (allerg* OR anaphyla* OR hypersensitivit*).” The timespan of the index publication date was set from January 1, 2013, to June 30, 2023. The literature type was restricted to articles or reviews, and the language was limited to English. A total of 13,534 articles were retrieved, and two authors individually manually screened the publications strongly related to drug hypersensitivity for improving accuracy. Conflicts were settled by discussion or by seeking the assistance of the third author. Finally, a total of 4299 relevant articles met the search criteria. The search strategy was shown in Supporting Information S1: Figure S1. The retrieved data were downloaded and exported in the form of all records and references and then manipulated as required by the CiteSpace for bibliometric and visual analysis.

### 2.2 Data analysis

CiteSpace was used to construct co‐occurrence analysis and visualize the collaboration networks of the authors/institutes/countries/keywords. Knowledge maps of author co‐citation analysis, reference co‐citation analysis, and burst keyword detection were also constructed. Different nodes represent different objects analyzed, such as countries/institutions/authors/keywords/cited references, and the size of the circle represents the number of publications and the color of the circle represents the year of their publications. The line between the two circles represents a cooperative relationship, and the thickness of the line represents the number/strength of cooperation. Moreover, Microsoft Excel 2016 was used to analyze the trend of the annual number of publications and growth. Additionally, we also use it to summarize the analysis results in the CiteSpace.

## 3 RESULTS

### 3.1 Bibliometric analysis of publication years and categories

**Figure 1:**
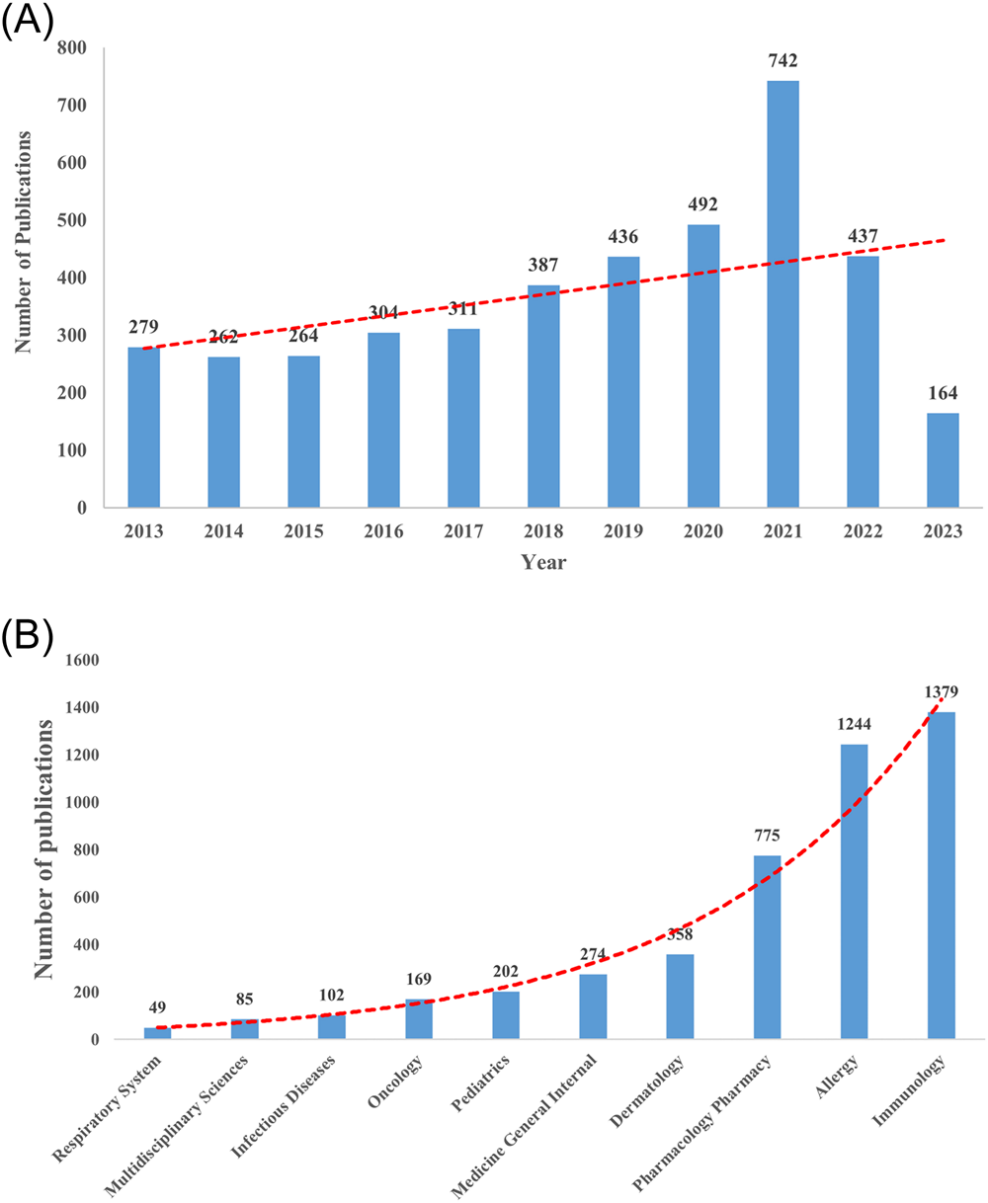
Trends in the number of publications and analysis of Web of Science Core Collection database (WoSCC) categories in the drug hypersensitivity. (A) The annual worldwide publication output. (B) WoSCC categories publication output.

The number of articles published in each period reflects the trend of research development in this field. As shown in Figure 1A, the number of research publications in the field of drug hypersensitivity generally increased over time, peaking in 2021 (*n* = 742).

**Figure 2:**
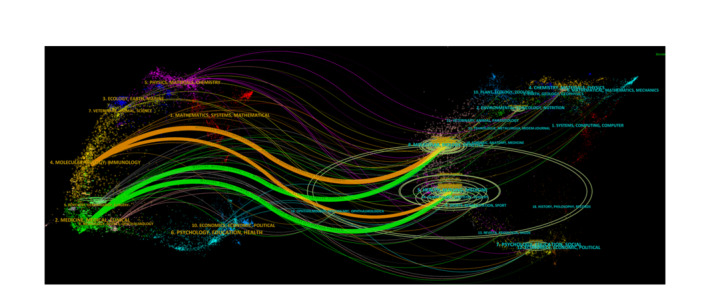
The dual‐map overlay of journals in the drug hypersensitivity.

The top 10 categories of drug hypersensitivity were presented in Figure 1B. Immunology was the most frequent category, with a frequency of 1379 publications, followed by Allergy with a frequency of 1244. Pharmacology Pharmacy (775 publications), Dermatology (358 publications), and Medicine General Internal (274 publications) were also remarkable categories. A dual‐map overlay was drawn to illustrate the relationship between drug hypersensitivity categories (Figure 2). The left hand was citing journals and the right hand was cited journals, whose colored paths between them suggested the interactions. There were four different citation paths. Two orange citation paths indicated that studies in the molecular/biology/immunology categories were mainly cited in the molecular/biology/genetics categories and health/nursing/medicine categories. Another two green citation paths indicated that studies in the medicine/medical/clinical categories were mainly cited in the molecular/biology/genetics categories and health/nursing/medicine categories.

### 3.2 Bibliometric analysis of countries and institutions

**Figure 3:**
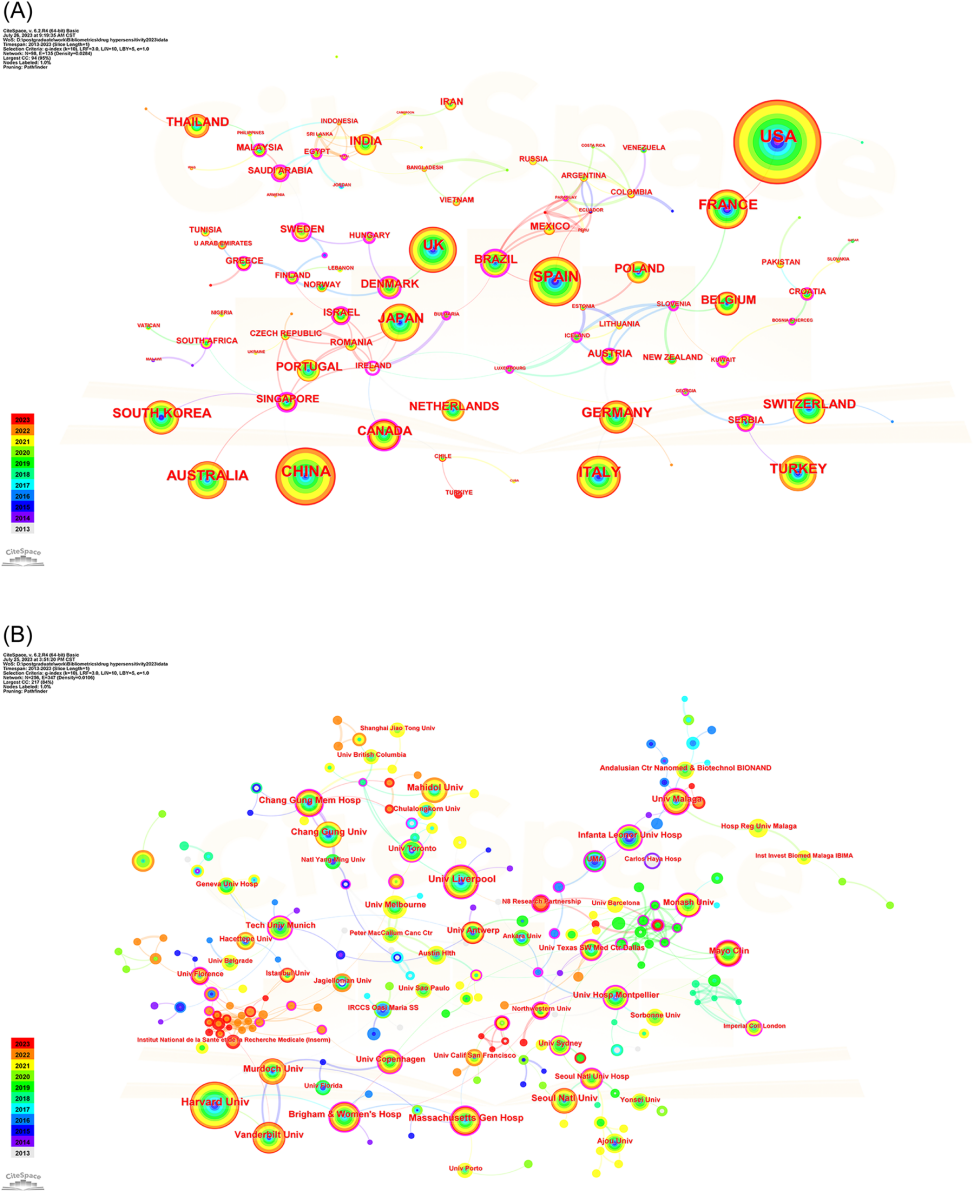
CiteSpace network visualization map of countries and institutions involved in the drug hypersensitivity. (A) Collaboration analysis of countries. (B) Collaboration analysis of institutions.

The country co‐occurrence knowledge map with a density of 0.0284 had 98 nodes and 135 edges (Figure 3A). The United States contributed the largest nodes and cooperated well with France. Additionally, despite having the second‐largest number of publications, China had less cooperation with other countries. The country marked with purple circles shows the strongest centrality score. With 10 cooperating countries, Ireland had the widest purple circle. Subsequently, the top 10 countries were listed in Table 1. The United States led the 98 countries with 1055 publications, followed by China (*n* = 521), Spain (*n* = 392), United Kingdom (*n* = 324), and Italy (*n* = 304).

**Table 1:** Top 10 countries and institutions related to drug hypersensitivity.

Rank	Country	Count	Centrality	Institution	Count	Centrality
1	USA	1055	0.04	Harvard University (USA)	183	0.07
2	China	521	0.00	University of Liverpool (UK)	92	0.12
3	Spain	392	0.01	Vanderbilt University (USA)	85	0.07
4	UK	324	0.00	Massachusetts Gen Hospital (USA)	77	0.12
5	Italy	304	0.00	Brigham & Women's Hospital (USA)	72	0.16
6	Australia	255	0.00	Murdoch University (Australia)	65	0.03
7	France	248	0.08	Chang Gung University (China)	63	0.00
8	Japan	241	0.02	Chang Gung Memorial Hospital (China)	63	0.14
9	Turkey	208	0.00	Seoul Natl University (Kore)	62	0.06
10	Germany	202	0.08	Mahidol University (Thailand)	62	0.05

The top 10 institutions published 824 articles, accounting for 19.17% of all articles published (Table 1). Harvard University ranked first (183 publications), followed by the University of Liverpool (92 publications), Vanderbilt University (85 publications), and Massachusetts Gen Hospital (77 publications). Brigham & Women's Hospital had the highest centrality (0.16) and contributed the fifth‐highest number of publications (*n* = 72). As shown in the Figure 3B, a collaboration between institutions was more comprehensive than that between countries.

### 3.3 Bibliometric analysis of authors and cited authors

**Figure 4:**
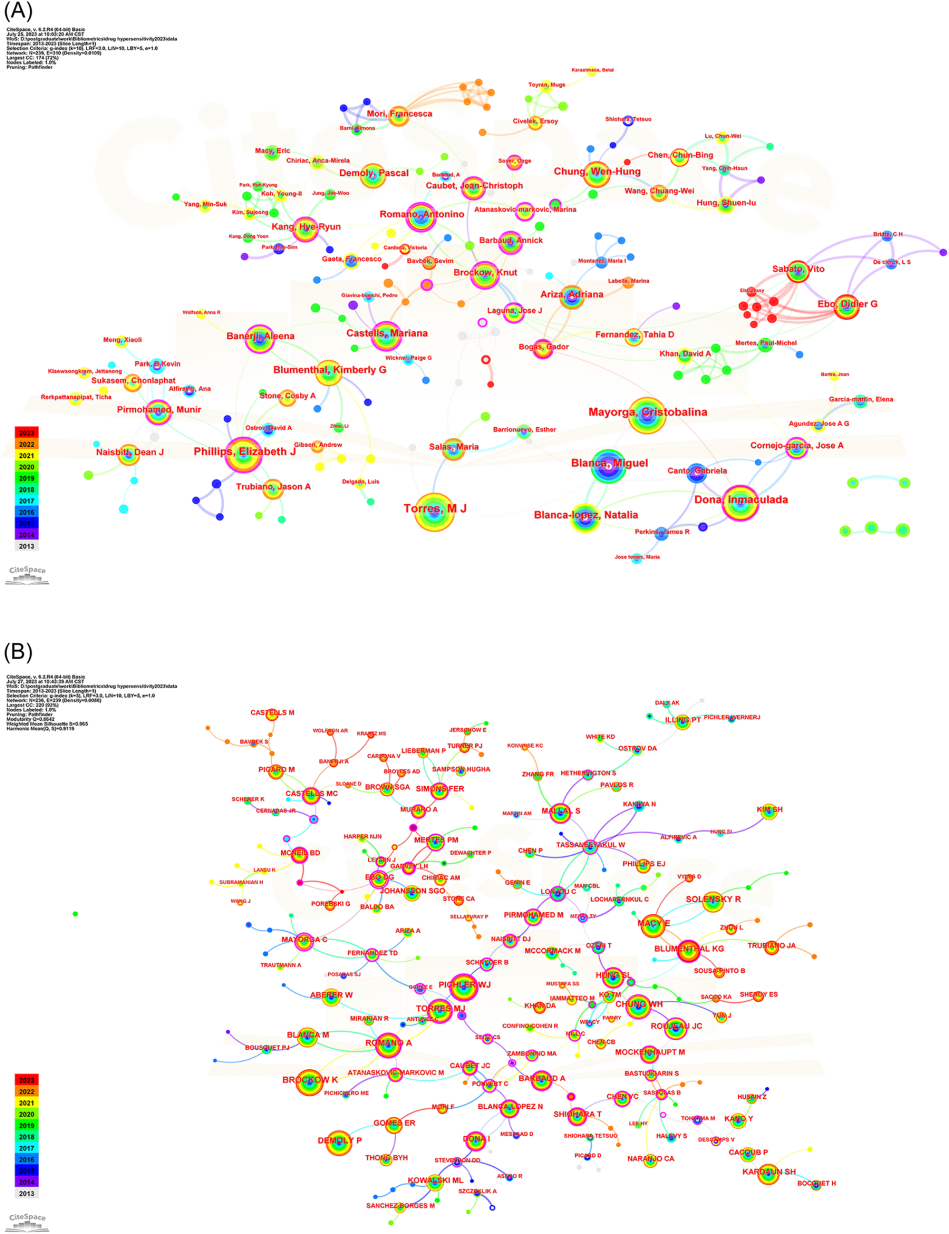
CiteSpace network visualization map of authors and co‐cited authors of publications related to drug hypersensitivity. (A) Collaboration analysis of authors. (B) Collaboration analysis of co‐authors.

The co‐occurrence knowledge map of authors comprised 239 nodes and 310 edges, with a density of 0.0109 (Figure 4A). As shown in Figure 4A, authors who published a large number of papers cooperated closely, whereas authors who published few papers did not. The top 10 authors contributed 618 papers. Five of the top 10 authors were from Spain, while the others were from China, United States, Australia, and France, respectively (Table 2). Torres from Spain was the most prolific author (*n* = 101), followed by Mayorga (*n* = 80), Dona (*n* = 73), Phillips (*n* = 66), Blanca (*n* = 65), Blanca‐Lopez (*n* = 53), Chung (*n* = 48), Blumenthal (*n* = 48), Castells (*n* = 44), and Demoly (*n* = 40).

**Table 2:** Top 10 authors related to drug hypersensitivity.

Rank	Authors	Country	Institution	Count	Centrality
1	Torres	Spain	The Institute of Biomedical Research of Málaga	101	0.03
2	Mayorga	Spain	The Institute of Biomedical Research of Málaga	80	0.00
3	Dona	Spain	University of Malaga	73	0.19
4	Phillips	Australia	Murdoch University	66	0.31
5	Blanca	Spain	Infanta Leonor University Hospital	65	0.02
6	Blanca‐lopez	Spain	Infanta Leonor University Hospital	53	0.02
7	Chung	China	Chang Gung Memorial Hospital	48	0.09
8	Blumenthal	USA	Harvard Medical School	48	0.07
9	Castells	USA	Harvard Medical School	44	0.43
10	Demoly	France	University of Montpellier	40	0.00

Co‐cited authors are two or more authors who are simultaneously cited by another or more publications, and these two or more authors comprise a co‐cited relationship. Brockow (595 publications), Demoly (515 publications), Pichler (494 publications), Romano (492 publications), and Macy (474 publications) were the most cited authors (Table 3). Demoly, Blanca, Chung, and Torres had the highest number of publications and citations. Figure 4B shows that the most productive authors typically had stable collaborations with other authors. Furthermore, an intimate co‐occurrence relationship between co‐cited authors and more productive authors, such as Pichler and Torres, was observed.

**Table 3:** The publisher did not receive permission from the copyright owner to include this object in this version of this product. Please refer either to the publisher's own online version of this product or the printed product where one exists.

### 3.4 Bibliometric analysis of journals and cited journals

A total of 1033 journals published articles on drug hypersensitivity. Table 4 presents the top 10 journals in drug hypersensitivity. The most productive journal was the Journal of Allergy and Clinical Immunology in Practice (215 publications), followed by Allergy (107 publications), Annals of Allergy Asthma and Immunology (80 publications), International Archives of Allergy and Immunology (79 publications), and Frontiers in Pharmacology (65 publications). Journal of Allergy and Clinical Immunology had the highest impact factor (IF) of 14.2 among the top 10 journals. Moreover, 30% of the journals were in Q1, 40% in Q2, and 30% in Q3. The powerfulness of journals is determined by the number of citations, which reflects whether the journal has an important influence in a particular research field. As shown in Table 4, nine journals have been cited more than 1000 times. Journal of Allergy and Clinical Immunology had the highest number of citation (2330), followed by Allergy (2150). Lancet had the highest IF of 168.9, followed by the New England Journal of Medicine with an IF of 158.5. Moreover, 60% of journals belonged to Q1.

**Table 4:** Top 10 journals and co‐cited journals related to drug hypersensitivity.

Rank	Journal	Count	IF (2023)	JCR	Cited‐Journal	Count	Centrality	IF (2023)	JCR
1	Journal of Allergy and Clinical Immunology‐In Practice	215	9.4	Q1	Journal of Allergy and Clinical Immunology	2330	0.29	14.2	Q1
2	Allergy	107	12.4	Q1	Allergy	2150	0.24	12.4	Q1
3	Annals of Allergy Asthma and Immunology	80	5.9	Q2	Annals of Allergy Asthma and Immunology	1562	0.02	5.9	Q2
4	International Archives of Allergy and Immunology	79	2.8	Q3	Clinical and Experimental Allergy	1487	0.09	6.1	Q2
5	Frontiers in Pharmacology	65	5.6	Q2	New England Journal of Medicine	1486	0.29	158.5	Q1
6	Journal of Allergy and Clinical Immunology	64	14.2	Q1	Journal of Allergy and Clinical Immunology‐In Practice	1387	0.09	9.4	Q1
7	Immunology and Allergy clinics of North America	53	2.6	Q3	Journal of Investigational Allergology and Clinical Immunology	1121	0.00	7.2	Q2
8	Current Opinion in Allergy and Clinical Immunology	53	2.8	Q3	Lancet	1086	0.00	168.9	Q1
9	Clinical and Experimental Allergy	50	6.1	Q2	British Journal of Dermatology	1039	0.05	10.3	Q1
10	Journal of Investigational Allergology and Clinical Immunology	50	7.2	Q2	International Archives of Allergy and Immunology	960	0.04	2.8	Q3

Abbreviation: IF, impact factor; JCR, journal citation reports.

### 3.5 Bibliometric analysis of references

References are an indispensable part of publications. The higher the frequency of citations, to a certain extent, the greater the influence of the publication is. Supporting Information S2: Table S1 summarizes the top 10 cited references in drug hypersensitivity. “International Consensus on Drug Allergy,” written by Demoly et al. and published in Allergy, was the most frequently cited article, followed by “Skin Test Concentrations for Systemically Administered Drugs ‐ an ENDA/EAACI Drug Allergy Interest Group position paper” and “Health Care Use and Serious Infection Prevalence Associated with Penicillin Allergy in Hospitalized Patients: A Cohort Study.” Allergy and Nature had a tremendous scientific impact on researchers and academics in drug hypersensitivity. Furthermore, all journals belonged Q1 among the top 10 references.

**Figure 5:**
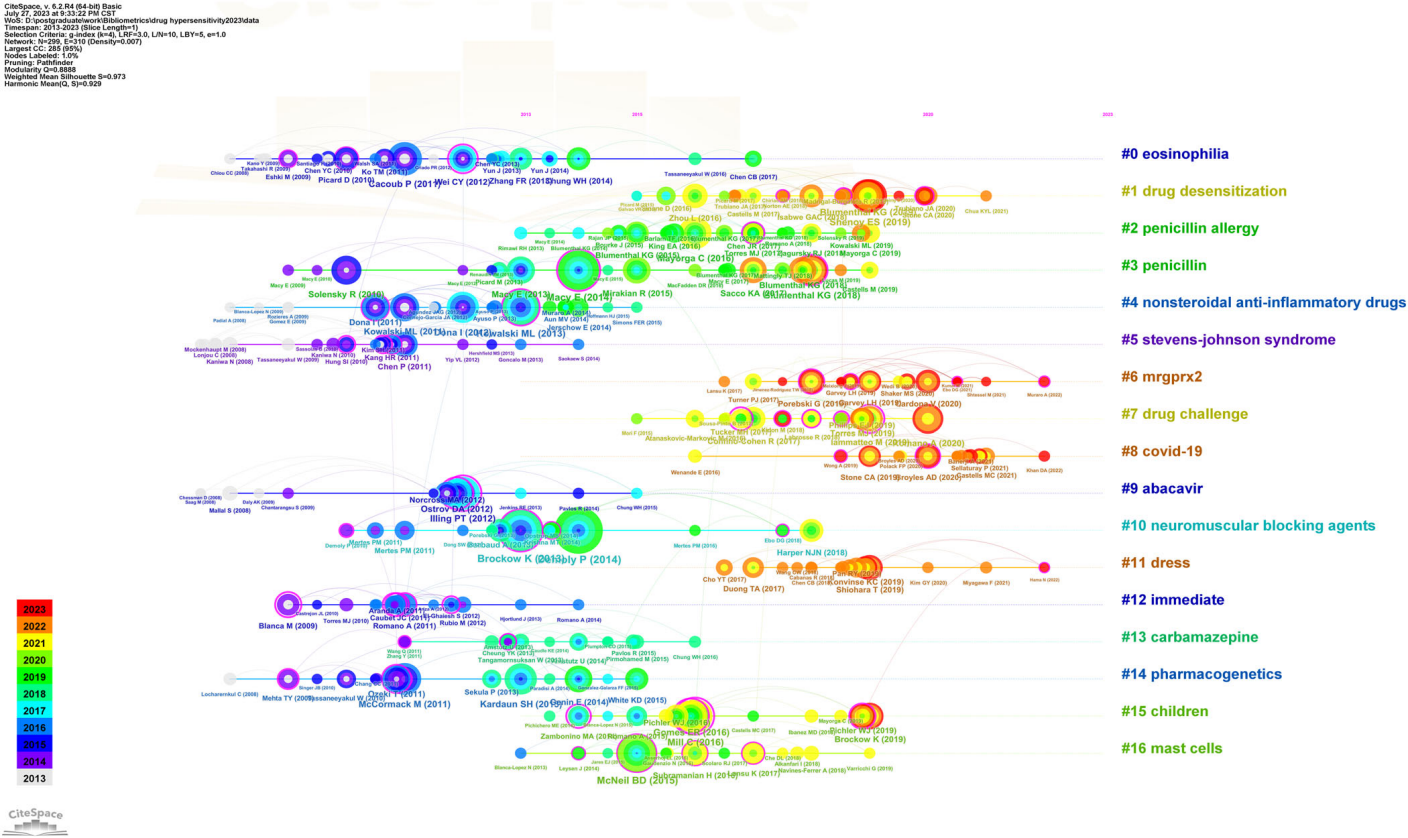
Timeline viewer of references from publications on the drug hypersensitivity.

A timeline viewer of references and extracted cluster labels from keywords was built (Figure 5). The largest cluster was eosinophilia (#0), followed by drug desensitization (#1), penicillin allergy (#2), penicillin (#3), nonsteroidal anti‐inflammatory drugs (#4), stevens‐johnson syndrome (#5), mrgprx2 (#6), drug challenge (#7), COVID‐19 (#8), abacavir (#9), neuromuscular blocking agents (#10), and dress (#11). Clusters #0, #3, and #14 (pharmacogenetics) were always the hotspots of drug hypersensitivity. Moreover, Clusters #6, #8, and #11 had been emerging hotspots since 2016.

### 3.6 Bibliometric analysis of keywords

**Figure 6:**
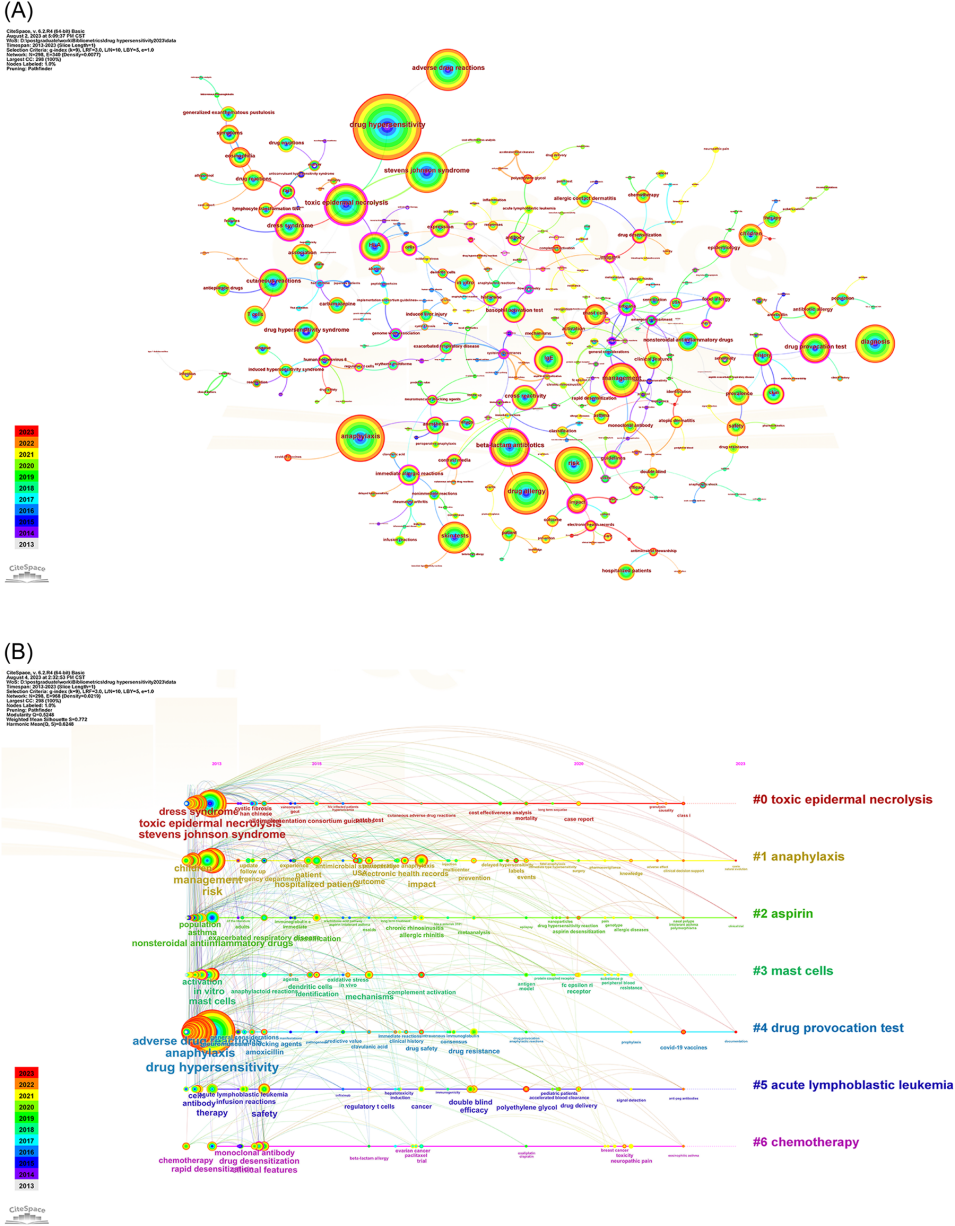
CiteSpace visualization map of keywords related to the drug hypersensitivity. (A) Keyword co‐occurrence network. (B) Timeline viewer of keywords.

Keywords are the kernel of a publication. By analyzing the keywords, we can summarize research topics in a specific field and explore hotspots and research directions. A keyword co‐occurrence knowledge map was employed (Figure 6A). The map was composed of 286 nodes and 314 edges, and the density was 0.0077. The top 20 keywords were listed in Table 5. Excluding keywords related to drug hypersensitivity (drug hypersensitivity, anaphylaxis, adverse drug reactions, and drug allergy), the keywords appearing more than 300 times in this study were stevens johnson syndrome (535), toxic epidermal necrolysis (498), diagnosis (473), beta‐lactam antibiotics (460), risk (449), management (387), and skin tests (324), indicating that these keywords were the hotspots of drug hypersensitivity.

**Table 5:** Top 20 keywords related to drug hypersensitivity.

Rank	Keywords	Count	Centrality	Rank	Keywords	Count	Centrality
1	drug hypersensitivity	1334	0.01	11	skin tests	324	0.03
2	anaphylaxis	712	0.01	12	dress syndrome	260	0.28
3	adverse drug reactions	572	0.00	13	drug provocation test	258	0.23
4	drug allergy	564	0.00	14	HLA	245	0.44
5	stevens johnson syndrome	535	0.01	15	IgE	241	0.11
6	toxic epidermal necrolysis	498	0.40	16	cutaneous reactions	224	0.11
7	diagnosis	473	0.00	17	cross reactivity	209	0.12
8	beta‐lactam antibiotics	460	0.21	18	children	184	0.08
9	risk	449	0.00	19	drug hypersensitivity syndrome	180	0.08
10	management	387	0.13	20	mast cells	173	0.09

A timeline viewer was constructed based on the interaction and mutation of keywords in a specific field. The timeline viewer, shown in Figure 6B, visually showed the phased hotspots and future directions of drug hypersensitivity from the time dimension. The timeline viewer produced seven clusters, including toxic epidermal necrolysis, anaphylaxis, aspirin, mast cells, drug provocation test (DPT), acute lymphoblastic leukemia, and chemotherapy.

**Figure 7:**
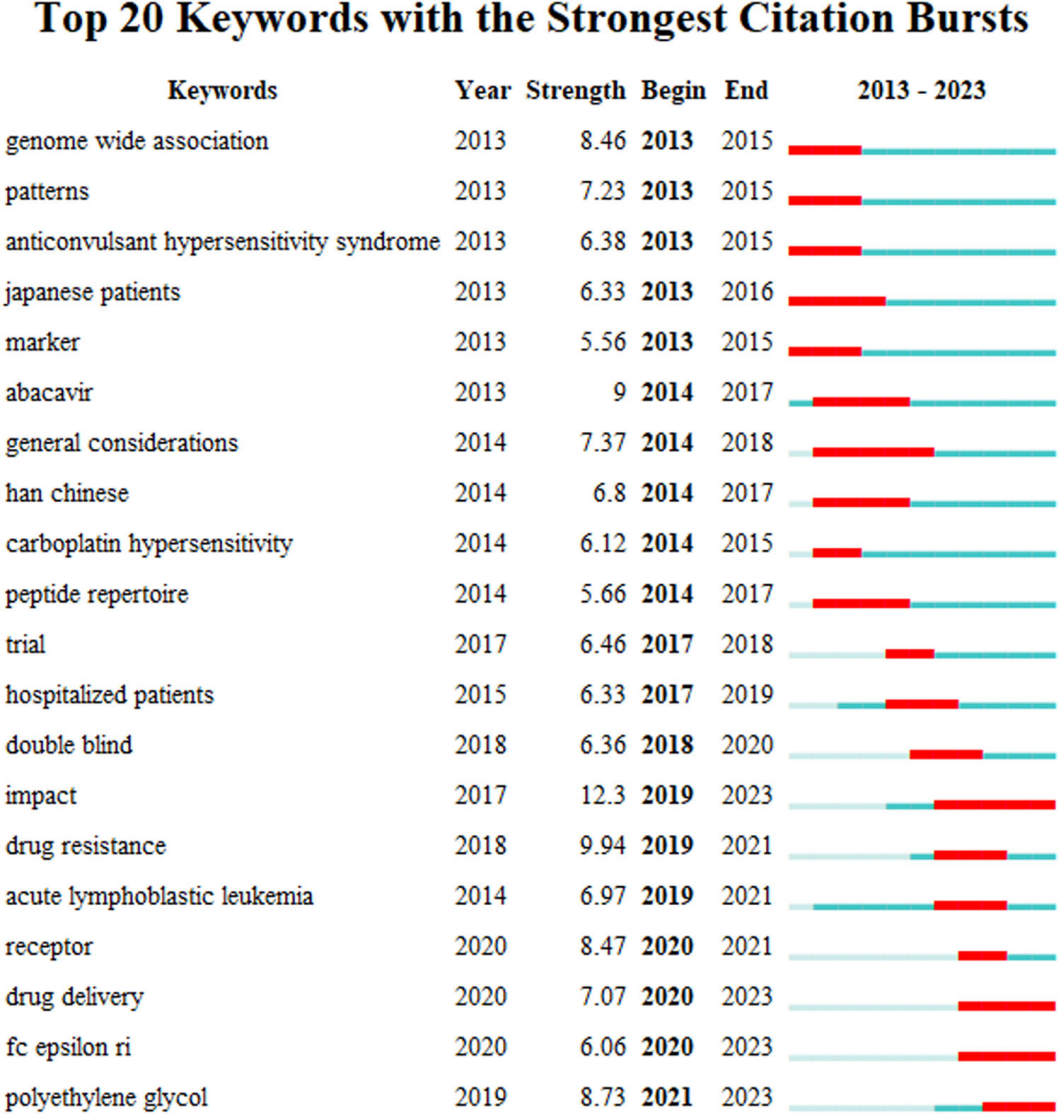
Top 20 keywords with the strongest citation bursts involved in drug hypersensitivity.

Keyword burst detection refers to keywords that appear frequently in a specific period, enabling us to explore the evolutionary process and characteristics of drug hypersensitivity. A map of the top 20 keywords with the strongest citation bursts was generated (Figure 7), in which the blue part indicating the time interval, and the red part indicating the duration period when a keyword had a burst. The leading burst keywords in the early stage (2013–2016) were genome‐wide association, pattern, anticonvulsant hypersensitivity syndrome, Japanese patients, and marker. In the middle stage (2014–2019), abacavir, general considerations, Han Chinese, carboplatin hypersensitivity, peptide repertoir, trial, and hospitalized patients were the research hotspots. Recently (2020–2023), double blind, impact, drug resistance, acute lymphoblastic leukemia, receptor, drug delivery, fc epsilon ri, and polyethylene glycol had become novel burst words.

## 4 DISCUSSION

A bibliometric analysis of the publications on drug hypersensitivity from 2013 to 2023 via CiteSpace is performed. The number of annual publications on drug hypersensitivity has gradually increased over the selected decade and appears to be a sharp increase in 2021. We speculate that the coronavirus disease 2019 (COVID‐19) outbreak causes more people to become ill and require medication, resulting in more hypersensitivity reactions. From the perspective of countries contribution, the United States ranks first in the world, indicating that the United States has become the most significant contributor to drug hypersensitivity. China is the only developing country, however, it accounts for the second highest publications. Moreover, most of the articles are published within the past 4 years. This could be explained by the fact that China has a high prevalence of drug hypersensitivity. Yet, China has a relatively low centrality, which indicates that Chinese researchers should broaden their transnational collaboration and information exchange to increase their influence.

Torres is the most productive author, who plays an important role in different types of drug hypersensitivity and participates in writing the drug hypersensitivity interest group position paper and developing innovative methods of hypersensitivity classification, diagnosis, and management.^11^, ^12^, ^13^ Furthermore, Torres and his cooperators will allow researchers to better understand the potential mechanisms of drug hypersensitivity.^14^ Chung is the most productive and co‐cited Chinese researcher, who concentrates on genetic variants associated with cytotoxic T lymphocyte (CTL)‐mediated drug hypersensitivity.^15^, ^16^, ^17^ Chung et al. recently identified that HLA‐B*13:01 is closely related to co‐trimoxazole‐induced drug hypersensitivity in Asians.^18^ Additionally, Chung et al. propose that monitoring CTL‐mediated bullous skin diseases via granulysin level is a noninvasive, rapid, and useful tool to differentiate other bullous skin diseases.^19^ Obviously, the tight cooperation map is between Chung and other Chinese researchers, while the research scholars abroad are far away. This finding indicates that scholars at home and abroad should strengthen their cooperation and share information on drug hypersensitivity.

References with citation bursts explain how frequently research is cited during a certain period (Supporting Information S1: Figure S2). The most cited reference “International Consensus on drug allergy” with the strongest burst is published by Demoly from University of Montpellier. Demoly et al.^5^ highlight the same key information as many existing guidelines, meanwhile criticizing any differences or deficiencies, thus guiding the diagnosis and management of drug hypersensitivity. Notably, six articles with the citation bursts higher than 18 are published in the past 5 years. Shenoy,^20^ Romano^21^, and Blumenthal^22^ and colleagues provide a global update on antibiotic allergy epidemiology, classification, mechanisms, diagnosis, and management. Additionally, Shiohara et al.^23^ highlight several emerging views about the diagnosis, pathogenesis, and management of drug‐induced hypersensitivity syndrome (DIHS)/drug reaction with eosinophilia and systemic symptoms (DRESS) and Konvinse et al.^24^ recommend that HLA‐A*32:01 testing could help pre‐empt and implicate vancomycin‐induced DRESS.

Keywords can reflect the topical subject and central content of research. Hence, keyword co‐occurrence analysis may help to understand the research hotspots and future directions. Integrating keywords with the strongest citation burst and timeline viewer clusters, the research hotspots and development frontiers in the field of drug hypersensitivity are determined. The main contents are as follows.

### 4.1 Emerging causes

With the constant development of new drugs, the high incidence and mortality of drug hypersensitivity have been a global health issue until now. According to the Food and Drug Administration (FDA) Adverse Event Reporting System, antibiotics, nonsteroidal anti‐inflammatory drugs (NSAIDs), chemotherapy agents, and intraoperative agents are commonly associated with drug hypersensitivity.^25^ Here, we identify an emerging trend of increasing reports of anaphylaxis to polyethylene glycols (PEGs) over time, especially after the COVID‐19.

PEGs are excipients in numerous medications, health care products, cosmetics, and foods,^26^ and they are also in COVID‐19 vaccinations against the COVID‐19 outbreak.^27^ The incidence of allergy to COVID‐19 vaccinations is estimated at 7.91 cases per million doses.^28^ Hypersensitivity to PEG has increased because it is suspected to be one of the possible causes of COVID‐19 vaccination‐related allergic reactions.^29^ There is now great interest in learning more about this rare allergy. However, the evidence supports that PEG of anaphylaxis to COVID‐19 vaccinations is contradictory and the cause may be multifactorial.^30^ Although the exact mechanism of allergic reactions anaphylaxis to COVID‐19 vaccinations remains unknown, it is necessary to elucidate the mechanism and exchange knowledge between PEG and COVID‐19 vaccinations. An algorithm that uses a stepwise approach of skin prick test (SPT) to PEG of increasing molecular weights and concentrations to diagnose PEGs allergy.^27^

### 4.2 Potential mechanisms

Drugs are considered to be foreign antigens. Understanding the potential mechanisms is critical in deciding whether to readminister or avoid. The variabilities in mechanisms may be concerned with chemical structure, size, and bio‐distribution of culprit drugs, which could explain the different drug sensitization, rare clinical manifestations, dose dependence, predictability, and cross reactivity.^31^

Human leukocyte antigen (HLA), a component of the human major histocompatibility complex, is the first keyword related to the mechanism. Four hypothesizes have been proposed that HLA is key regulator of T‐cell‐mediated drug hypersensitivity: (i) the “hapten” theory,^32^ (ii) the “p‐i” concept,^33^ (iii) the “altered peptide repertoire” model,^34^ and (iv) the “altered T‐cell receptor (TCR) repertoire” model.^35^ For example, patients with carbamazepine hypersensitivity may be associated with the public αβTCR on T cells that trigger immune responses against HLA‐B*15:02‐presented drug antigens.^36^ Another study shows that programmed cell death protein 1 inhibitory pathway and CD4 T‐cell depletion both contribute to abacavir hypersensitivity.^37^ Notably, different HLAs have mechanism differences in the same/different drugs and constitute a bridge between pharmacology and pharmacogenetics for personalized treatment.

IgE is the second mechanically related keyword and fc epsilon ri (FcεRI) has the strongest burst citation in our study. Currently, the pathway for IgE‐mediated drug hypersensitivity is well described.^38^ IgE‐mediated drug hypersensitivity is contributed to drug‐sIgE through covalently binding to mast cells or basophils with FcεRI based on the “hapten” theory.^39^ Jang et al.^40^ show that crosslinking Cnidium officinale Makino‐sIgE stimulated mast cell activation through extracellular singal‐regulated Kinases (ERK)/nuclear factor kappa‐light‐chain‐enhancer of Activated B Cells (NF‐kB) pathway. However, some new mechanisms of IgE‐mediated drug hypersensitivity emerge when considering the different dynamics of forming noncovalent drug–protein complexes or covalent hapten–protein adducts^41^: (i) reduced mast cell reactivity; (ii) noncovalent drug–protein complexes called “fake antigens” with cross‐link preformed drug‐sIgE; (iii) a metabolic step for forming a reactive metabolite. Hence, more studies should be consequently prompted to confirm or disapprove the new mechanisms.

MAS‐related G‐protein coupled receptor‐X2 (MRGPRX2), a specific receptor responsible for pseudo‐allergic reactions determined by Johns Hopkins University,^42^ which is cited from 2017 to 2021 based on the timeline viewer of references. MRGPRX2 can regulate mast cell degranulation by influencing intracellular Ca^2+^ concentration via phosphatidylinositol 3 kinase (PI3K)/serum‐threonine kinase (AKT) and phospholipase C gamma (PLCγ) signaling^43^ and MRGPRX2‐SOCE‐STIM1 pathway in mast cells would resolve the treatment of pseudo‐allergic reactions in humans.^44^ Whether it is IgE or non‐IgE‐mediated drug hypersensitivity, some limitations of mechanisms in humans' drug hypersensitivities exist on account of the rarity and unpredictability of drug hypersensitivity. Hence, evidence from animal models on the mechanisms of anaphylaxis could provide an edified knowledge of various mechanisms. Moreover, precise phenotypic definitions and high‐throughput platforms are needed to elucidate these mechanisms.

### 4.3 Clinical diagnosis

The diagnosis of drug hypersensitivity aims to provide a safety guarantee for patients' clinical medication and reduce risk. Moreover, diagnosis is extremely complicated and highly dependent on the mechanisms involved.^45^ From a practical perspective, drug hypersensitivity diagnosis can be performed by clinical history combined with in vivo and in vitro tests depending on the type of reactions (IDHR and NIDHR).^5^, ^46^

A detailed medical history of drug hypersensitivity is the first step for allergy specialists to evaluate. The skin is the most common and prominent organ influenced by drug hypersensitivity, with the most severe forms manifesting as stevens‐johnson syndrome and toxic epidermal necrolysis (SJS/TEN),^47^ which are the top keywords appeared in our study. Additionally, DIHS/DRESS is a potentially lethal multiorgan hypersensitivity reaction relative to the reactivation of human herpesvirus 6, which has been attracted the attention of scholars by 2022. Currently, the diagnosis of DIHS/DRESS remains challenging due to possible delays in diagnosis and variability in presentation, course, and severity.^48^ However, these manifestations do not represent a confirmed drug hypersensitivity, just only a history of anaphylaxis after drug exposure. Despite its limitations, the medical history is an important starting point for selecting the next steps and classifying responses into IDHR and NIDHR, as well as conducting a risk assessment to guide further investigations.

In vivo tests are the second step in diagnosing drug hypersensitivity. They refer to a method of re‐exposing patients to culprit drugs and observing various clinical manifestations in patients. The vivo tests including skin tests (STs) and DPTs are the high‐frequency keywords. Whether hypersensitivity reaction is IgE‐mediated or not, STs involving intradermal test (IDT) and SPT are of great importance in evaluating drug hypersensitivity reactions.^49^, ^50^ For IDHR, the workup typically includes SPT and immediate‐reading IDTs.^11^ In a patient with a history of an IDHR, no positive irritation at drug concentrations suggests an enhanced sensitivity for drug‐sIgE.^51^ Moreover, they appear to be determined for IDHR to β‐lactam antibiotics, perioperative drugs, platin salts, and heparins, but moderate to low for most other drugs.^52^ In the case of NIDHR, there are still concerns regarding the safety of STs in triggering a severe reaction, the diagnostic procedure starts with a patch test (PT) and if negative, to continue with SPT and IDT.^53^, ^54^, ^55^ PT applies only to non‐IgE‐mediated hypersensitivity reactions, especially in delayed hypersensitivity to drugs.^50^ Unfortunately, most drug allergens have limited true diagnostic value. Standardized and validated test concentrations and vehicles have not been well studied or are controversial in the literature.^51^ Additionally, high heterogeneity of ST in which NIDHR exists in different countries.^53^ The use of IDT and PT for diagnosing is not routinely performed in the United States due to the FDA lacking detection reagents and specialty centers,^56^ while European guidelines recommend applying PT and IDT.^11^ Moreover, Chinese researchers suggest PT is safe and effective in carbamazepine‐induced drug hypersensitivity.^57^ Another Australian researcher demonstrates the combined use of in vivo tests (PT and IDT) and ex vivo test in NIDHR to antibiotics has high specificity.^58^ Importantly, improving patient compliance with STs to reduce recurrent DHR is necessary. Moreover, standardized methodological approaches, on the other hand, are lacking, and concentration is inconsistent. Hence, the appropriate drug concentration must be adjusted based on the patient's skin condition, drug type, and local guidelines. DPT is the gold standard for identifying culprit drugs,^5^ which can further be conducted when clinical history, STs, and sIgE determinations are negative.^49^ However, DPT cannot distinguish between IDHR and NIDHR because it is not associated with pathogenesis.^5^ DPT can be performed by oral, nasal, inhaled, or intravenous route, with the oral route being the primary method. A retrospective analysis for eliciting dose thresholds (reactive doses) in beta‐lactam hypersensitivity has confirmed the safety of the 1‐day protocol for both IDHR and mid‐NIDHR.^59^ Caubet et al.^60^ suggest that follow‐up DPT in children with NIDHR seems to be safe and may improve the diagnosis. Although DPT is superior to other STs, it is a risky procedure and has some limitations. First, a heterogeneity exists in DPT protocols among countries and among centers within the same country. Second, due to the risk of a possible serious reaction when reintroducing the suspected drug, confirmatory DPTs cannot be performed for ethical reasons.^61^ Third, the patient is unwilling to be re‐exposed to a drug that he or she considers pernicious. What is more, noncontrollable and/or severe life‐threatening drug hypersensitivity cannot be conducted DPT. Finally, a negative test does not demonstrate future tolerance to the drug, but the time of the challenge and the doses challenged are low.^5^ Further studies are necessary to provide data on the standardization of protocols for DPT, particularly NIDHR.

In vitro test is a biological test performed on a patient's blood or other types of biological material outside the body, which are available for characterizing DHRs depending on the underlying mechanism and reaction phase.^46^ IDHR may involve different mechanisms: IgE‐mediated or off‐target mechanisms.^62^ Measurements of histamine^63^ and tryptase^64^ levels have been shown to help confirm acute IgE‐mediated hypersensitivity reactions. Notably, single tryptase or histamine results can only indicate that the culprit drug can stimulate the release of tryptase or histamine, and cannot absolutely illustrate the causal relationship between sensitizing drugs and drug hypersensitivity. sIgE determination whose clinical application is unclear is based on a solid phase detection of drug‐sIgE in serum with drug‐carrier conjugate.^65^ Nowadays, only a few drugs sIgE are currently available with the positive rate from 26% to 92%. For the beta‐lactams hypersensitivity, measurement of sIgE has been used to assess cross‐reactivity, however, its clinical application is unclear.^65^ Basophil activation test (BAT), a method for diagnosing IgE‐mediated reactions using flow cytometry with sensitivity from 55% to 80% and specificity from 80% to 96%,^66^ had been active until now based on the timeline viewer of references. Additionally, BAT may be the only diagnostic method addressable, which is often a cheaper and securer alternative to aforementioned tests.^66^ Yet, only beta‐lactams,^67^ neuromuscular‐blocking drugs,^68^ NSAIDs,^69^ quinolones,^70^ iodinated contrast media,^71^ and vaccination^72^ are appropriate. In 2023, Mayorga et al. conducted a practical investigation into BAT use and utility in IDHR and developed a position paper with detailed recommendations.^73^ The HLA genotyping with a high frequency can effectively detect specific drugs in susceptible people. The HLA‐A*32:01 variant has been highlighted as a diagnostic biomarker for vancomycin hypersensitivity in European populations,^24^ and the HLA‐B*13:01 variant may dramatically decrease the incidence of dapsone hypersensitivity in Chinese populations.^74^ HLA‐B*15:02 relates to carbamazepine hypersensitivity in Chinese population, while HLA‐A*31:01 is found in Northern European populations.^5^ Regardless, identifying relationships between HLA, drugs, and populations allows us to more precisely diagnose and treat. Certainly, the in vitro test also includes lymphocyte transformation test (LTT), enzyme‐linked immunospot essay (ELISpot), and intracellular cytokine staining (ICS). LTT, based on the proliferative response of T‐lymphocytes under stimulation with the culprit drug, has been widely used to evaluate T‐cell‐mediated DHR or NIDHR.^63^ A recent study including 31 patients with suspected hypersensitivity to Benznidazole shows an overall sensitivity of 80% and a specificity of 82%.^75^ Bellon and colleagues suggest that LTT can be useful in evaluating drug causation SJS/TEN patients taking multiple medications.^76^ One way to improve the sensitivity of NDHR in vitro test is to evaluate the mechanism of effect.^46^ The results of interferon γ (IFN‐γ)‐releasing cells by ELISpot are detectable in almost half of patients with drug‐induced severe cutaneous adverse reactions.^77^ Compared with LTT and ELISpot, ICS has higher sensitivity in detecting interleukin‐5 (IL‐5) and IFN‐γ, which can improve the diagnostic sensitivity of SJS/TEN.^78^ Although different assays have been implemented to test more drugs to determine their sensitivity and specificity, most are still not available for clinical practice. Among all in vitro tests, only HLA allele screening can have diagnostic value for a few specific drugs. Therefore, we can easily conclude from these that there is no approach with optimal/adequate sensitivity for diagnosing drug hypersensitivity. With the rapid development of computer technology, various artificial intelligence algorithms have emerged as new tools for massive data mining and analysis. Inglis et al.^79^ construct an artificial neural network and implement risk stratification of penicillin allergy. Sharma et al.^80^ use random forest to develop a computational tool for predicting the allergenicity of chemical compounds. Further research is needed to develop a truly safe test that can definitively diagnose DHR.

According to the timeline viewer of references, drug desensitization has always been a focus of scholars. Drug desensitization can be performed for rational clinical use after the culprit drug confirmed by in vivo or in vitro tests. When culprit drugs are the only effective drugs for the current treatment of underlying diseases, drug desensitization should be adopted.^81^ Nowadays, no accepted or official protocol for desensitization of each drug exists. European Academy of Allergy and Clinical Immunology suggests starting with 1/1,000,000–1/10,000 of the therapeutic dose and gradually increasing 10 steps to avoid severe reactions.^82^ A study of drug desensitization for chemotherapy shows that rapid drug desensitization represents the best approach to maintain cancer patients with antineoplastic drugs hypersensitivity on their most effective treatments.^83^ Moreover, desensitization has been shown to be useful to in iron product allergy,^84^ NSAIDs,^85^ antibiotics,^86^ and so on.

Bibliometric analysis based on CiteSpace provides a better insight into global trends, however, our study has several limitations. First, only publications other than the English language from WOSCC are obtained, which may occur some bias. Even so, the number of publications selected is much enough to represent the current mapping of drug hypersensitivity. Second, the quality of publications in WOS is not identical, and a weighted analysis of publications based on quality assessment is beyond the scope of our study. Still, our analysis gives the same attention to publications of different quality. Third, though the data have been manually normalized, errors may exist since the author with the same name or keywords of various expressions. Despite this, we tried to include as many similar authors or keywords as possible.

Generally, research on drug hypersensitivity is in a rapid development stage. CiteSpace knowledge mapping provides a deeper insight of the evolutionary path, frontier hotspots, and future direction over the last decade. Scholars and countries' cooperations are characteristics and trends in the field of drug hypersensitivity. Additionally, an emerging trend in reports of anaphylaxis to PEG is identified. Future perspectives should focus on the development of algorithms for understanding the standardization process according to the culprit drug, clinical manifestations, and diagnostic methods. Moreover, a better understanding of the mechanisms to culprit drugs with immunological precise phenotypic definitions and high‐throughput platforms is needed.

## AUTHOR CONTRIBUTIONS

Cairong Gao and Xiangjie Guo conceived the idea for the study. Li Luo, Niannian Chen, and Zhanpeng Li collected the data. Li Luo, Cairong Gao, and Xiangjie Guo discussed the relation of data. Zhanpeng Li, Chunmei Zhao, Yiming Dong, Likai Wang, Xiaoqian Li, Wenchao Zhou, and Yingna Li analyzed the data. Li Luo and Niannian Chen wrote the manuscript. Cairong Gao and Xiangjie Guo revised and approved the final version of the manuscript. All authors contributed to the article and approved the submitted version.

## CONFLICT OF INTEREST STATEMENT

The authors declare no conflict of interest.

## Supporting information

Supporting information.

Supporting information.

## Data Availability

The datasets used and analyzed during the current study are available from the corresponding author on reasonable request.
