# Supplementary material for: Knowledge mapping and global trends of drug hypersensitivity from 2013 to 2023: A bibliometric analysis
Source: Immun Inflamm Dis. 2024 Apr 17;12(4):e1245. doi: 10.1002/iid3.1245 (PMC11022627; doi:10.1002/iid3.1245)
Supplement: Supplementary file 1 — Supporting information. [file IID3-12-e1245-s001.docx]

Supplementary Material

# Supplementary Figures and Tables

## Supplementary Figures


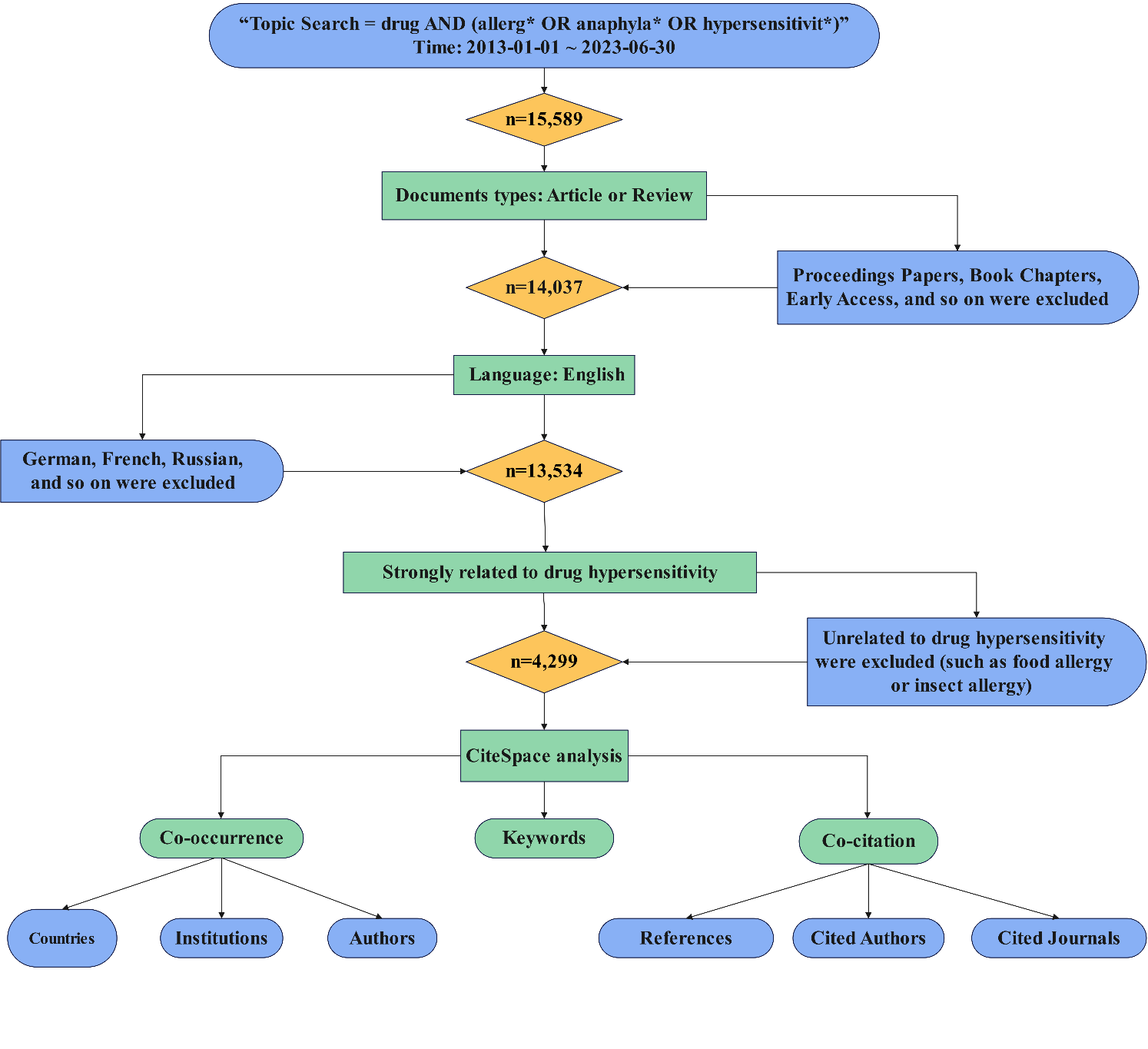


Figure S1. Flowchart of literature selection.


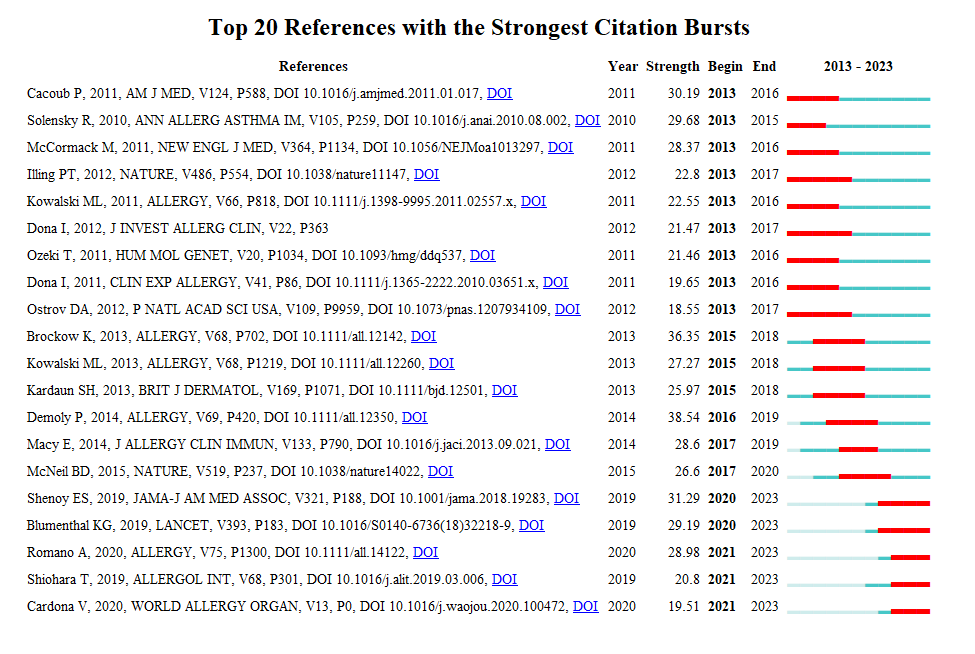


Figure S2. Top 20 references with the strongest citation bursts involved in drug hypersensitivity.

## Supplementary Table

**Supplementary Table 1.** Top 10 co-cited references related to drug hypersensitivity.
